# High accessory pathway conductivity blocks antegrade conduction in Wolff‐Parkinson‐White syndrome: A simulation study

**DOI:** 10.1002/joa3.12528

**Published:** 2021-03-24

**Authors:** Ryo Haraguchi, Takashi Ashihara, Taka‐aki Matsuyama, Jun Yoshimoto

**Affiliations:** ^1^ Graduate School of Applied Informatics University of Hyogo Kobe Japan; ^2^ Center for Information Technology and Management Shiga University of Medical Science Otsu Japan; ^3^ Department of Legal Medicine School of Medicine Showa University Tokyo Japan; ^4^ Department of Pediatric Cardiology Shizuoka Children's Hospital Shizuoka Japan

**Keywords:** accessory pathway, antegrade conduction, computer simulation, Wolff‐Parkinson‐White syndrome

## Abstract

**Background:**

Wolff‐Parkinson‐White (WPW) syndrome is characterized by an anomalous accessory pathway (AP) that connects the atrium and ventricles, which can cause abnormal myocardial excitation and cardiac arrhythmias. The morphological and electrophysiological details of the AP remain unclear. The size and conductivity of the AP may affect conduction and WPW syndrome symptoms.

**Methods:**

To clarify this issue, we performed computer simulations of antegrade AP conduction using a simplified wall model. We focused on the bundle size of the AP and myocardial electrical conductivity during antegrade conduction (from the atrium to the ventricle).

**Results:**

We found that a thick AP and high ventricular conductivity promoted antegrade conduction, whereas a thin AP is unable to deliver the transmembrane current required for electric conduction. High ventricular conductivity amplifies transmembrane current. These findings suggest the involvement of a source‐sink mechanism. Furthermore, we found that high AP conductivity blocked antegrade conduction. As AP conductivity increased, sustained outward transmembrane currents were observed. This finding suggests the involvement of an electrotonic effect.

**Conclusions:**

The findings of our theoretical simulation suggest that AP size, ventricular conductivity, and AP conductivity affect antegrade conduction through different mechanisms. Our findings provide new insights into the morphological and electrophysiological details of the AP.

## Graphical abstract

We performed computer simulations of antegrade accessory pathway (AP) conduction using a simplied wall model. We found that high AP conductivity blocked antegrade conduction. This finding suggests the involvement of an electrotonic effect.

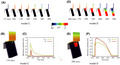

## 1 INTRODUCTION

Accessory pathways (APs) are microscopic anomalous muscular bundles that connect the atrial and ventricular myocardium, bypassing the normal conduction system. APs can cause abnormal excitation of the myocardium leading to cardiac arrhythmias.^1^ APs are present in patients with Wolff‐Parkinson‐White (WPW) syndrome.^2^ Clinically, WPW syndrome is classified according to the location of the APs, as established by electrocardiographic (ECG) and electrophysiological tests.^3^, ^4^ Recently, radiofrequency AP catheter ablation has been used to treat WPW syndrome with good results.^5^, ^6^

However, the morphological and electrophysiological details of APs remain unclear. Few studies have investigated the morphological and electrophysiological characteristics of APs.^7^, ^8^ Wei et al^9^ reproduced the ECG characteristics of WPW in a computer simulation. Recently, we successfully visualized APs using three‐dimensional (3D) reconstruction images of histological sections.^10^

It is widely accepted that a narrow AP poorly sustains electrical conduction.^11^ Myocardial electrical conductivity affects the stability of excitation propagation. Preliminary computer simulations of AP conduction were performed using a simplified 3D wall model to investigate the effects of the morphological and electrophysiological properties of AP on conduction. We focused on the effects of AP bundle size and myocardial electrical conductivity on antegrade (from atrium to ventricle) AP conduction. A preliminary version of this work has been reported.^12^

## 2 METHODS

**FIGURE 1:**
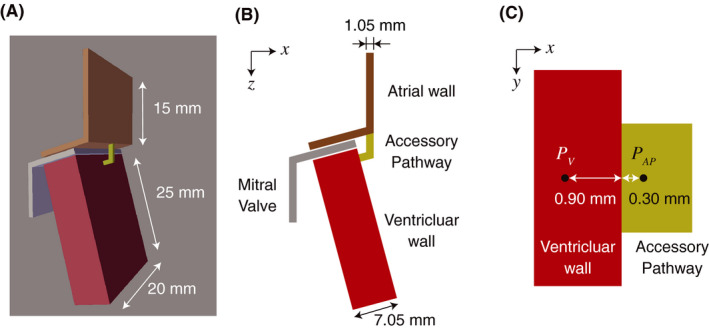
The simplified three‐dimensional model featuring an atrial and ventricular wall and a myocardial bundle. A, Three‐dimensional view. The atrial wall (brown) and the ventricular wall (red) are connected by the accessory pathway (dark‐yellow). The mitral valve (gray) is disconnected electrically. B, Cross‐sectional view. C, Enlarged view near the junction of the ventricular wall and accessory pathway. Black dots indicate the sites where membrane potentials and currents were recorded in the simulation study. This figure is revised from (c) Ryo Haraguchi et al (2019)^1^/CC‐BY‐4.0

We constructed a 3D model consisting of a simplified atrial wall, ventricular wall, and myocardial bundle representing an AP (Figure 1A,B). The atrial model combined two walls 20 × 15 × 1.05 and 20 × 10 × 1.05 mm in dimension. The ventricular wall dimensions were 20 × 25 × 7.05 mm. Although the left ventricular free wall thickness is usually approximately 10 mm, the thickness was set to 7.05 mm in our model because the myocardium near the mitral valve is generally thin. The myocardial bundle models (AP models), which included various bundle sizes, were integrated with the atrial and ventricular models. We constructed five models using various bundle sizes (Table 1).

**TABLE 1:** Configuration of the accessory pathway in the simplified three‐dimensional models

Name	Accessory pathway cross‐section size (mm)	Contact area between the accessory pathway and ventricle (mm^2^)
Model A	0.6 × 0.6	0.54
Model B	0.75 × 0.75	0.68
Model C	0.9 × 0.9	0.95
Model D	1.05 × 1.05	1.42
Model E	1.95 × 1.05	2.63

To investigate AP conduction in detail, we configured recording sites near the junction of the AP (*P*_AP_) and ventricular wall (*P*_V_, Figure 1C) to record membrane potential and current during the simulation experiments.

The membrane kinetics of the simulated atrial myocardium and AP were produced using Courtemanche‐Ramirez‐Nattel mathematical equation.^13^ The membrane kinetics of the simulated ventricular myocardium were produced using O’Hara‐Rudy model equation^14^ with modification of the conductance of the sodium channel current. The electrophysiological models were Hodgkin‐Huxley type differential equation,^15^ which were configured based on the experimentally measured responses of various ion channel/exchange/pump currents. Furthermore, the models incorporated intracellular calcium dynamics and extracellular/intracellular ion concentrations. The wet experiments validated whole‐cell responses such as stimulus frequency dependence of the action potential duration (APD) as well as early afterdepolarizations (EAD) and APD alternans.

To simulate the propagation of a cardiac action potential, cardiac tissue is usually modeled as a two‐phase ohmic medium (one phase representing the intracellular space and the other representing the extracellular space) with the two phases linked by a network of resistors and capacitors.^16^ Monodomain equation^17^ were used as governing equations in our 3D model. The no‐flux Neumann boundary condition was applied to the tissue border. We configured six levels of isotropic intercellular conductivity, ranging from 0.17 to 1.36 mS/cm. Previous studies have shown that conduction velocity can be achieved within a physiological range at these conductivity levels.^12^, ^18^, ^19^

To simulate excitation conduction from the atrium to the ventricle via the AP, 10 pacing stimuli (2‐ms duration and 2,000 pA amplitude) were delivered to the top of the atrial wall at a cycle length of 1,000 ms After the last pacing stimulus, we measured membrane potentials and transmembrane currents at the *P*_AP_ and *P*_V_ recording sites.

The simulation program was written in C++ with OpenMP for parallel processing. We used the forward Euler numerical integration method to solve the ordinary differential equations numerically. In all the simulations, the spatial resolution per unit was set to 0.150 mm in all directions, and the time step was set to 0.005 ms

## 3 RESULTS

### 3.1 A thick AP triggers antegrade conduction

**FIGURE 2:**
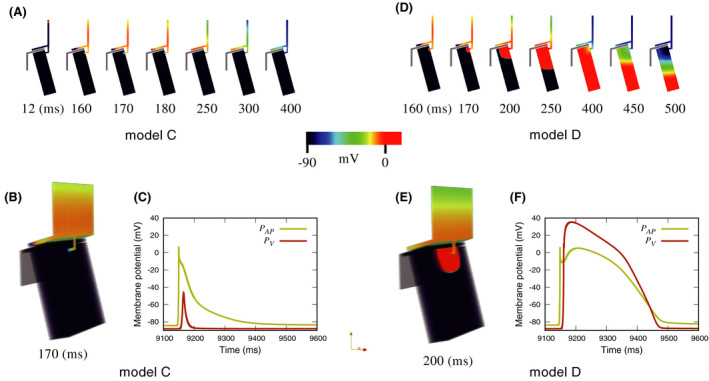
Examples demonstrating the effect of section size of the accessory pathway in the simplified three‐dimensional models, (A, D) cross‐sectional view of antegrade conduction; (B, E) three‐dimensional view of antegrade conduction; and (C, F) effects of section size of the accessory pathway on membrane potential morphology at the *P_AP_* and *P_V_* recording sites. This figure is revised from (c) Ryo Haraguchi et al (2019)^1^/CC‐BY‐4.0

**FIGURE 3:**
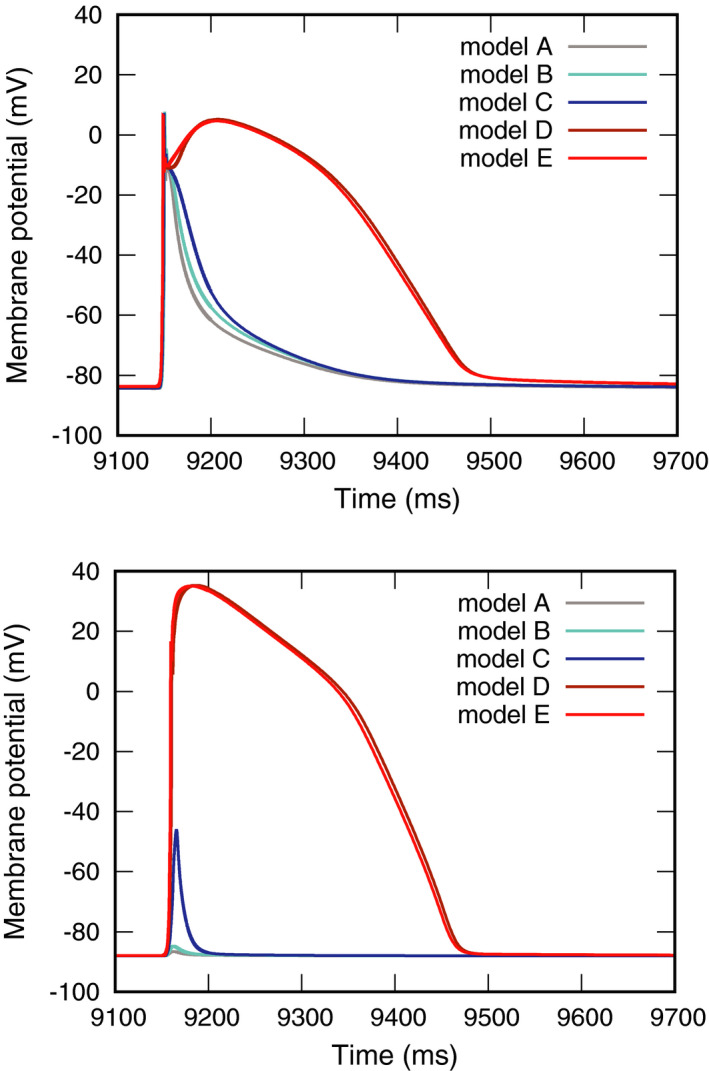
Effects of accessory pathway cross‐section size. Membrane potentials at the *P*_AP_ (top) *P*_V_ (bottom) recording sites

First, we set the isotropic intercellular conductance to 0.17 mS/cm in the atrial and ventricular walls and the AP. Under constant intercellular conductance, the excitation propagation ran from the atria to the AP, but not from the AP to the ventricle in model C (Figure 2A,B). Moreover, no action potentials were generated at the *P*_AP_ and *P*_V_ recording sites (Figure 2C). In contrast, in model D, in which the AP was thicker, excitation propagation ran from the atria to the ventricle via the AP (Figure 2D,E), and normal action potentials were recorded at *P*_AP_ and *P*_V_ (Figure 2F). AP thickness had an effect on membrane potential (Figure 3). No action potentials were generated in models A–C, in which the APs were thinner. In contrast, the action potentials were normal in models D and E with thicker simulated APs.

**FIGURE 4:**
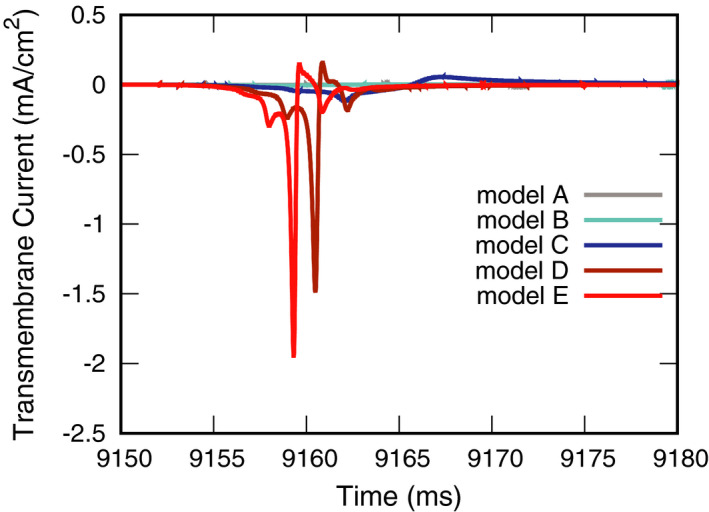
Effects of accessory pathway cross‐section size on transmembrane currents at the *P*_V_ recording site

Furthermore, AP bundle size had an effect on the transmembrane current (Figure 4). The transmembrane currents recorded at *P*_V_ were larger in models D and E than in models A–C. Moreover, the peak time was slightly earlier in model E than in model D.

These findings suggest that a thick AP triggered antegrade conduction. In other words, a small bundle blocked conduction.

### 3.2 Ventricular conductivity promotes antegrade conduction

In model C, where the intercellular conductivity was 0.17 mS/cm, excitatory propagation from the AP to the ventricle was blocked (Figure 2). In contrast, with the higher ventricular conductivity (*g*_V_ = 0.20 mS/cm), there was excitatory propagation from the atria to the ventricle via the AP.

**FIGURE 5:**
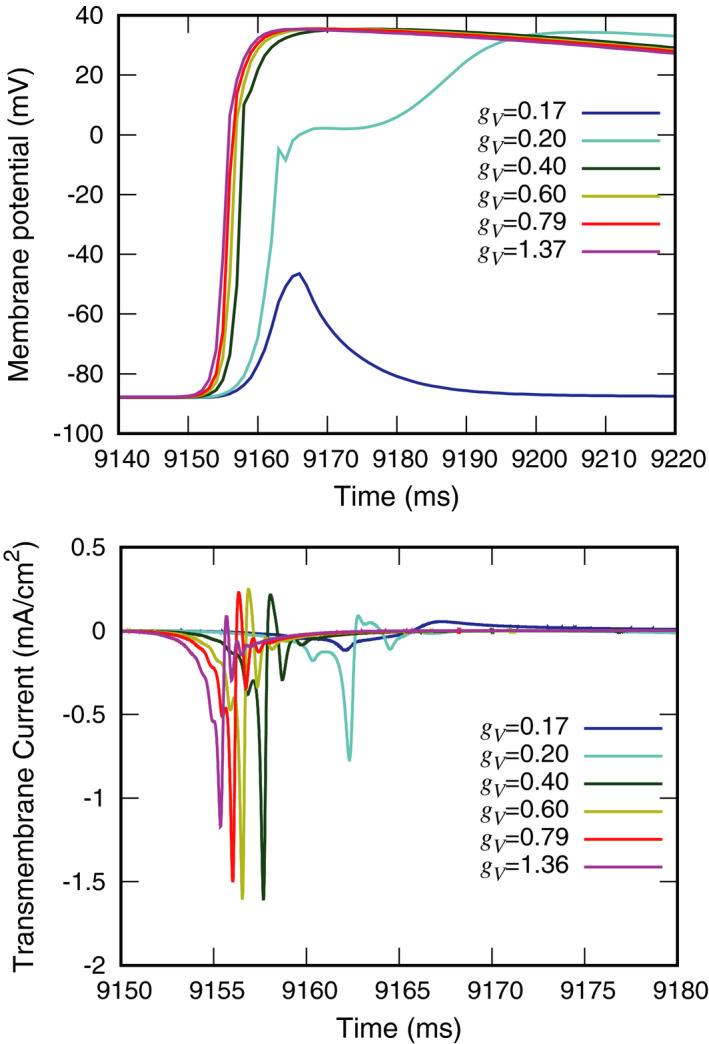
Effects of ventricular conductivity on membrane potentials (top) and transmembrane currents (bottom) at the *P*_V_ recording site in model C

Figure 5 top shows the effect of ventricular conductivity on membrane potentials in model C. Normal action potentials were recorded in model C with higher ventricular conductivity. Gradual depolarization with a slight delay in peak time was recorded with *g*_V_ = 0.20 mS/cm.

Figure 5 bottom shows the effect of ventricular conductivity on transmembrane current in model C. At *g*_V_ = 0.20 mS/cm, the peak current was lower, and the peak time was slightly earlier, than with *g*_V_ = 0.40, 0.60, 0.79 and 1.36 mS/cm. These findings suggest that ventricular intercellular conductivity promotes antegrade conduction.

### 3.3 AP conductivity prevents antegrade conduction

When the intercellular conductivity was 0.17 mS/cm in model D, excitation propagation ran from the atria to the ventricle via the AP (Figure 2). In contrast, high AP conductivity (*g*_AP_ = 0.79 and 1.36 mS/cm) blocked conduction from the AP to the ventricle.

**FIGURE 6:**
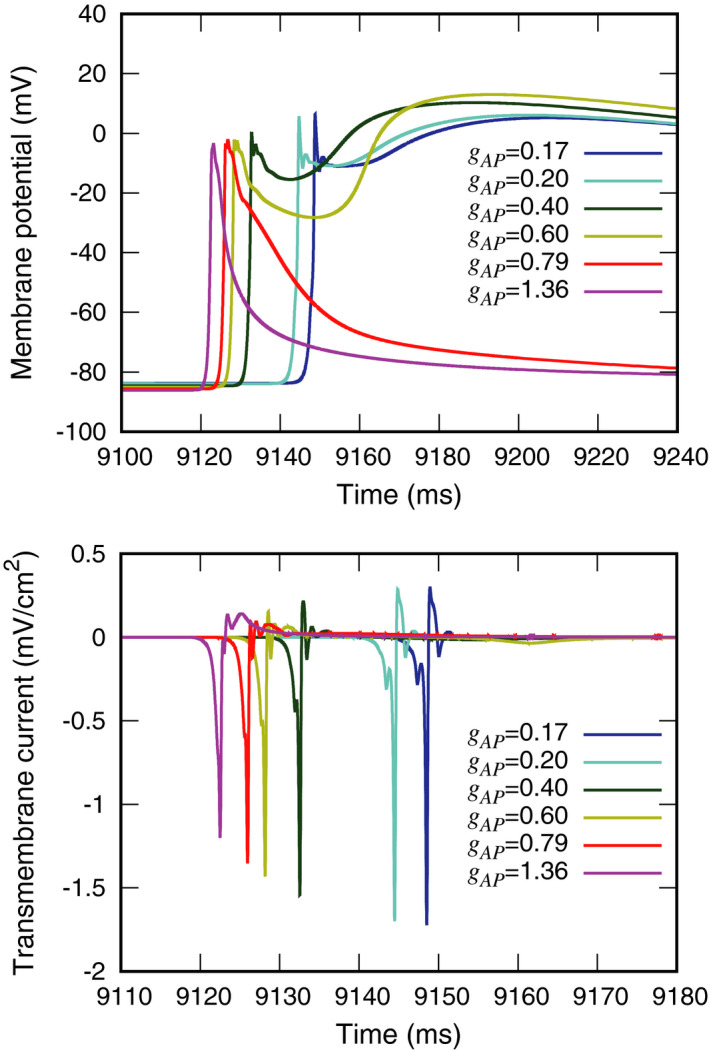
Effects of accessory pathway conductivity on membrane potentials (top) and transmembrane currents (bottom) at the *P*_AP_ recording site

Figure 6 top shows the effect of AP conductivity on the membrane potentials in model D. Incomplete action potentials were observed in model D, which simulated high AP conductivity. However, the peak potentials elicited by the various conductivity levels were not significantly different.

Figure 6 bottom shows the effect of AP conductivity on transmembrane currents in model D. Under the *g*_AP_ = 0.79 and 1.36 mS/cm conditions, the outward current durations were longer than those recorded at lower AP conductivity.

As AP conductivity increased, complete action potentials were not formed, suggesting that AP conductivity blocked antegrade conduction.

## 4 DISCUSSION

We investigated the effects of AP bundle size and intercellular conductivity on antegrade conduction and provided new insights into the mechanisms underlying WPW syndrome. Our analysis of the morphological and electrophysiological properties of AP revealed that thick AP and high ventricular conductivity promoted antegrade conduction through source‐sink relationship, and high AP conductivity blocked antegrade conduction through an electrotonic effect.

### 4.1 Effects of AP bundle size and ventricular conductivity on antegrade conduction

We investigated whether antegrade conduction from the atrium to the ventricle passed through an AP. Antegrade conduction was blocked when the AP bundle was small but occurred when the bundle was large. When we increased ventricular conductivity in a small AP bundle, antegrade conduction occurred. Both AP bundle size and ventricular conductivity had marked effect on the peak inward transmembrane current. These findings can be explained by a source‐sink relationship.^11^, ^20^ Spector^21^ described the electric propagation in terms of the source of the depolarizing current and the tissue (sink) to be depolarized.In our study, the AP bundle provided a smaller source than the sink of the larger ventricular tissue sink to which it was connected. Therefore, the source‐sink balance was asymmetrical. A small AP would be unable to supply the current required for electric conduction. As the AP bundle size increased, the total amount of transmembrane current at the junction of the AP and ventricular wall increased. As ventricular conductivity increased, the transmembrane current density at the junction of the AP and ventricular wall increased. From the perspective of source‐sink balance, both increased the "source". Our findings clarified the roles of AP bundle size and ventricular conductivity in antegrade AP conduction.

### 4.2 Effect of AP conductivity on antegrade conduction

Antegrade conduction was observed in model D. However, when AP conductivity was increased, antegrade conduction was blocked. Differences in AP conductivity did not affect the peak membrane potential or inward transmembrane current. In contrast, sustained outward transmembrane currents were recorded when antegrade conduction was blocked, suggesting an electrotonic effect.^22^ Myocardial tissues interact with each other electrically. The extent of such interactions depends on the membrane potential difference and intercellular conductivity. As AP conductivity increases, the electrotonic current rises in both the waveback area and the wavefront. These findings indicate that the sustained outward current prevented formation of the action potential waveform despite having sufficient current for excitation propagation. From the source‐sink balance perspective, increased AP conductivity provided a larger sink via an increase in the outward transmembrane current.^21^ These findings clarify the effect of AP conductivity on antegrade AP conduction.

### 4.3 Comparison with previous studies

de la Fuente et al^23^ reported that conduction was blocked at the junctions of a narrow band of tissue communicating between two larger areas in isolated canine atrial tissue. Inoue et al^24^ reported that conduction was depressed over a narrow isthmus of atrial myocardium in open‐chested dogs. Our finding that antegrade conduction was blocked in simulations with small AP bundles is consistent with that of Inoue et al.

The excitation propagation proceeded from the atrium to the AP in all simulations. Antegrade conduction was blocked near the junction of the AP and ventricular wall in models with a thin AP, low ventricular conductivity, and high AP conductivity. Kuck et al^25^ used multipolar electrode catheters to systematically record AP activation, to identify sites of antegrade and retrograde conduction block in 126 patients. They found that antegrade AP conduction was most often blocked at or near the AP‐ventricular interface. Our simulations are consistent with these findings.

### 4.4 Physiological and clinical implications

Wood et al^26^ provided the first histological evidence of APs. Arruda et al^4^ developed an ECG algorithm to estimate AP location, which has been used clinically for ablation treatment of WPW syndrome. However, few studies have investigated the histological or electrophysiological properties of APs in depth.^10^, ^27^ Previous computer simulations have been limited to the reproduction of ECG waveforms,^9^, ^28^ whereas our simulations demonstrated the effects of AP bundle size and intercellular conductivity on AP conduction. Simulation studies require the integration of various morphological and electrophysiological parameters into a computer model. Because these parameters are not well defined for the AP, our findings provide useful information for building a simulation model to study the mechanism underlying paroxysmal supraventricular tachycardia (PSVT, atrioventricular reentrant tachycardia [AVRT]).

APs, such as the Kent bundle, are congenital in nature; however, symptomatic WPW in patients with normal hearts is age dependent.^29^ We found that reduced AP conductivity triggered anterograde conduction. In general, myocardial conductivity decreases with age. Our simulation findings suggest that an age‐related reduction in AP conductivity leads to the onset of WPW syndrome, which may be suppressed by using drug treatment to alter AP conductivity.

### 4.5 Study limitations

We did not consider ion channel conductance in our atrial wall, ventricular wall, or AP models. Furthermore, we used the Courtemanche‐Ramirez‐Nattel model^13^ to reproduce the membrane kinetics of the AP; however, the model was developed to reproduce human atrial action potentials. Few studies have investigated the electrophysiological properties of ion channel conductance in APs. Ion channel conductance is thought to influence the excitability and refractoriness of AP conduction.

Furthermore, we did not explore retrograde conduction. Gallagher et al^1^ and Tonkin et al^27^ showed that the AP refractory period was markedly different in anterograde and retrograde conduction. An AP with a short effective refractory period is associated with a high risk of sudden cardiac death in patients with WPW syndrome.^29^, ^30^ We focused on the AP bundle size and myocardial conductivity, and thus, did not investigate the refractory period of the AP during atrial fibrillation.

We used a simplified 3D wall model and did not investigate the effects of AP shape or length, heterogeneity, or anisotropy. The electrophysiological properties and shape at the junction of the AP and ventricular wall may affect AP conduction.

## 5 CONCLUSIONS

The findings of our theoretical simulation suggest that AP size, ventricular conductivity, and AP conductivity affect antegrade conduction through different mechanisms. We offer new insights into the morphological and electrophysiological details of the AP.

## DISCLOSURES

Authors declare no Conflict of Interests for this article.
